# Phylogenetic Revision of the Genus *Aliivibrio*: Intra- and Inter-Species Variance Among Clusters Suggest a Wider Diversity of Species

**DOI:** 10.3389/fmicb.2021.626759

**Published:** 2021-02-18

**Authors:** Terje Klemetsen, Christian R. Karlsen, Nils P. Willassen

**Affiliations:** ^1^Department of Chemistry, Center for Bioinformatics, UiT The Arctic University of Norway, Tromsø, Norway; ^2^Department of Fish Health, Nofima, Aas, Norway

**Keywords:** *Vibrionaceae*, *Aliivibrio*, phylogeny, multilocus sequence analysis, marine bacteria, species group coherence, marker gene

## Abstract

Genus *Aliivibrio* is known to harbor species exhibiting bioluminescence as well as pathogenic behavior affecting the fish farming industry. Current phylogenetic understanding of *Aliivibrio* has largely remained dormant after reclassification disentangled it from the *Vibrio* genus in 2007. There is growing evidence of wider diversity, but until now the lack of genomes and selective use of type strains have limited the ability to compare and classify strains firmly. In this study, a total of 143 bacterial strains, including 51 novel sequenced strains, were used to strengthen phylogenetic relationships in *Aliivibrio* by exploring intra-species and inter-species relations. Multilocus sequence analysis (MLSA), applying the six housekeeping genes *16S ribosomal RNA* (*rRNA*), *gapA*, *gyrB*, *pyrH*, *recA*, and *rpoA*, inferred 12 clades and a singular branch in *Aliivibrio*. Along with four new phylogenetic clades, the MLSA resolved prior inconsistencies circumscribing *Aliivibrio wodanis* and formed a unique clade we propose as the novel species *Aliivibrio* sp. “friggae.” Furthermore, phylogenetic assessment of individual marker genes showed *gyrB*, *pyrH*, and *recA* superior to the 16S rRNA gene, resolving accurately for most species clades in *Aliivibrio*. In this study, we provide a robust phylogenetic groundwork for *Aliivibrio* as a reference point to classification of species.

## Introduction

The family of *Vibrionaceae* contains a large number of bacterial species, many of which are described from marine habitats (Thompson et al., 2004). A comprehensive study of the family and its evolutionary history suggested a common ancestor dating back to the Devonian era some 600 million years ago (Sawabe et al., 2007). *Vibrionaceae* is versatile, delineated and holds 22 distinct phylogenetic clades with highly diverse species of which several are harbored within the genus *Aliivibrio* (Sawabe et al., 2007, 2013). *Aliivibrio* is a firmly established genus, separate from *Vibrio* (Ast et al., 2009; Boyd et al., 2015). The genus harbors bioluminescent bacteria that have symbiotic relationships with aquatic organisms (Bongrand and Ruby, 2019), but also includes pathogens of marine animals (Hjerde et al., 2015; Kashulin et al., 2017).

*Aliivibrio fischeri* is studied extensively for its bioluminescence and symbiotic capability with marine squids and fishes (Visick, 2009). Exploration of *A. fischeri* has focused on revealing and understanding mechanisms of host adaptation, biofilm formation, flagellar function, quorum sensing and subsequent pathways to express its observed phenotypes (Visick, 2009; Verma and Miyashiro, 2013). The ability to form bioluminescent symbiosis with marine hosts has additionally been observed in *Aliivibrio logei* and *Aliivibrio* sp. “thorii” (Ast et al., 2009), while *Aliivibrio sifiae* is capable of forming independent bioluminescent colonies on marine agar (Yoshizawa et al., 2010). *Aliivibrio logei* is associated to skin of farmed fish (Benediktsdóttir et al., 1998), but also found in shellfish (Bang et al., 1978) and in the intestine of fish residing in the seas of Bering and Okhotsk (Bazhenov et al., 2019). *Aliivibrio finisterrensis* has been isolated from free living clams (*Ruditapes philippinarum*) and shown to be seasonally present in the hindgut of Tasmanian farmed Atlantic salmon (Beaz-Hidalgo et al., 2010; Hatje et al., 2014). *Aliivibrio wodanis* is associated to ulcerative skin problems of farmed fish (Karlsen et al., 2014), and *Aliivibrio salmonicida* is the causative agent of the seasonal cold-water vibrosis (Egidius et al., 1986). Strains of *Aliivibrio* sp. “thorii,” *A. sifiae* and the non-luminescent *A. finisterrensis* have been given less attention. With the description of *A. finisterrensis* (Beaz-Hidalgo et al., 2010) and *A. sifiae* (Yoshizawa et al., 2010), the number of *Aliivibrio* species is currently six (Parte, 2018).

Utilization of the 16S ribosomal RNA (rRNA) gene marker-sequence is the prevalent method of inferring evolutionary relationships between taxa. However, 16S rRNA gene sequences may not provide interspecies resolution, and it is deemed a poor marker for resolving phylogenetically distinct species of *Vibrionaceae* (Sawabe et al., 2013; Ashok Kumar et al., 2020). Alternative marker genes are used to improve the resolution of species and the accuracy of classification in PCR based methods. Markers with a low degree of sequence conservation are favorable (Lan et al., 2016). The same study additionally found the *coaE* marker gene to phylogenetically mimic the genome wide amino acid identity in *Bacillus*. Other studies have applied the *fur* gene to further increase discriminatory power within *Vibrionaceae* (Machado and Gram, 2015), while *glnAI* has been proposed as an improvement for *Bifidobacteriaceae* compared to the 16S rRNA marker (Killer et al., 2020). Although single markers can provide phylogenetic resolution of species, a combination is often necessary to increase discriminatory power and expose monophyletic groups. This became evident following the use of multilocus sequence analysis (MLSA) reclassifying *Aliivibrio* (Urbanczyk et al., 2007), and in the identification of *Aliivibrio* sp. “thorii” (Ast et al., 2009). To further improve the accuracy of species identification, genome sequencing and genome-wide analysis were introduced (Konstantinidis and Tiedje, 2005). Indeed, genomic taxonomy suggests a correction of the whole *Vibrionaceae* family to change its parent order from the *Vibrionales* to the *Enterobacterales* (Parks et al., 2018).

As methods for more accurate classification has advanced, corrections of strains representative for species within *Aliivibrio* (formerly *Photobacterium* and later *Vibrio*) have occurred several times. For example, strain ATCC 15382, formerly classified as *Vibrio logei*, has later been suggested as a representative of *A. wodanis* (Ast et al., 2009). The same study additionally linked luminescent strain SR6 and SA12 to *A. wodanis*. However, bioluminescence in *A. wodanis* has not been described (Lunder et al., 2000; Hjerde et al., 2015). Furthermore, *A. sifiae* was published by introducing strain H1-1^T^ and H1-2 (Yoshizawa et al., 2010), while a set of 11 strains by Ast et al. (2009) were informally named as the “sifiae” clade. Without comparison to the described type strains there is no evidence for this classification.

Understanding the ecology and evolution of *Aliivibrio* requires robust and accurate genus- and species-level taxa. The present taxonomic classification results from representative type strains of selected species, eluding the species concepts of genomically coherent groups of microorganisms (Rosselló-Mora and Amann, 2001). The aim of the study was to firmly establish phylogenetic knowledge about the *Aliivibrio* genus with new sequenced data and to analyze marker genes for accurate classification of *Aliivibrio* species.

## Materials and Methods

### Samples and Data Preparation

In this study, marker genes from 143 bacterial strains obtained from in-house sequenced genomes and GenBank (Benson et al., 2017; RRID:SCR_004860; Supplementary Table 1) were used to inferring the evolutionary relationships. Among these, 134 were represented by *Aliivibrio* strains and for comparison with neighboring genera, four strains of *Vibrio* and *Photobacterium* were included. The outgroup was represented by *Photorhabdus luminescens* subsp. *laumondii* TT01^T^ in line with prior phylogenetic studies (Urbanczyk et al., 2007; Ast et al., 2009). The type strains of *A. finisterrensis* DSM 23419^T^ and *A. logei* ATCC 29985^T^, and 51 additional *Aliivibrio* isolates were genome-sequenced for this study. Strains and isolates sequenced in this study are available from the authors upon request.

For sequencing, *Aliivibrio* isolates, were revived from cryopreserved glycerol stocks and cultured in Luria-Bertani (LB) broth supplied with 3.5% w/v sodium chloride at 12°C. Genomic DNA was extracted using the Qiagen DNeasy blood and tissue kit protocol for Gram-negative bacteria. Sequencing libraries were prepared using the Nextera XT DNA Library Preparation Kit (Illumina) according to the manufacturer’s protocol. The fragment size distribution was verified to 500–1,000 bp using the Agilent 2100 Bioanalyzer System. Libraries were multiplexed and sequenced on an Illumina MiSeq instrument (RRID:SCR_016379), using either MiSeq Reagent kits v2 (500 cycles) or v3 (600 cycles), yielding an average of 3.8 million reads per bacterial isolate (Supplementary Table 2).

All sequence reads were quality controlled and each genome was *de novo* assembled using the CLC Genomics Workbench (RRID:SCR_011853) version 8.0.3. Briefly, paired-end reads were imported using the built-inn CLC pipeline removing failed reads. Reads were further quality trimmed with an ambiguous limit of 2 and a quality limit of 0.05 while reads shorter than 15 bases were removed. *De novo* assembly was performed with default parameters, auto-detecting paired distances and performing scaffolding. A cutoff for minimum contig length was set to 500 bp. On average, the *de novo* assembly gave 343 scaffolds (N50 of 62,646) with total assembly lengths between 3.7 and 5.2 Mb, and an average coverage of 247.43x (Supplementary Table 2).

The assembled genomes were annotated using Prokka (Seemann, 2014; RRID:SCR_014732) version 1.13 on the Galaxy platform (Afgan et al., 2016; RRID:SCR_006281) with a default parameter setting. Annotated genomes were screened for the 16S rRNA, *gapA* (P0A9B2), *gyrB* (P0A2I3), *pyrH* (P65933), *recA* (P65977), and *rpoA* (Q664U6) genes, and identified sequences extracted. Public sequence data under the *Aliivibrio* taxa (taxonomy ID 511678) which contained all six genes were gathered from GenBank. Locus tag identifiers from the 90 public strains used in this study are listed in Supplementary Table 1.

Sequences in each gene locus were aligned individually using MUSCLE (Edgar, 2004; RRID:SCR_011812) version 3.8.31 with default parameters for nucleotide sequences. Gene regions were selected according to Sawabe et al. (2007). However, flanking ends in each alignment were recursively trimmed. Briefly, flanking positions with gaps occurring in more than 5% of the alignment sequences were trimmed using Aliview (Larsson, 2014; RRID:SCR_002780) version 1.2.6. *Vibrio cholerae* strain N16961 (ungapped gene numbering) was used as reference to the trimmed alignments with the following gene name, gene position range, and reference locus tag: 16S rRNA, 252-1422, and VCr001; *gapA*, 89-862, and VC2000; *gyrB*, 308-1496, and VC0015; *pyrH*, 21-624, and VC2258, *recA*, 69-865 VC0543; and *rpoA*, 20-950, and VC2571. The concatenated sequence of the six trimmed fragments (16S rRNA-*gapA-gyrB-pyrH-recA-rpoA*) produced a multilocus sequence alignment (MLSA) of 5,473 positions.

### Analysis

Phylogenetic relationships between bacterial strains included in this study were constructed on the basis of the concatenated MLSA. A network graph was created in SplitsTree4 (Huson and Bryant, 2005; RRID:SCR_014734) version 4.13.1 by applying the Jukes-Cantor (JC69) distance correction between sequences while NeighborNet was used as network model.

**Figure 1:**
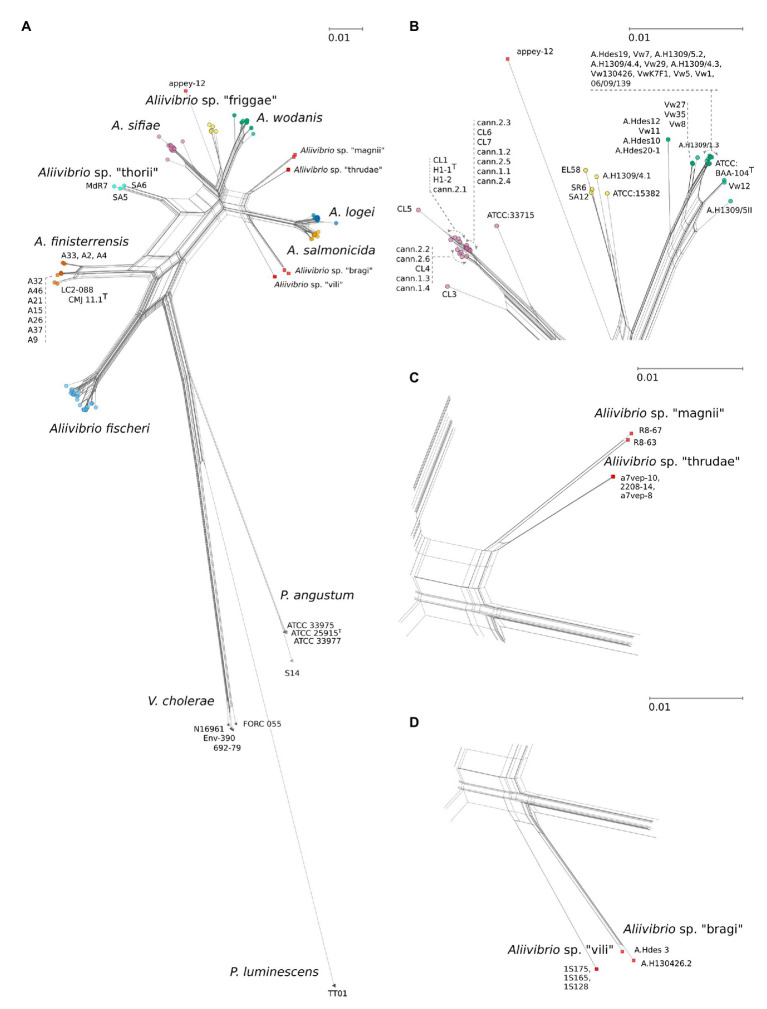
**(A)** JC69 corrected NeighborNet comprising 143 strains including *Aliivibrio*, *Vibrio cholerae*, *Photobacterium angustum*, and with *Pluminescens luminescens* acting as outgroup. Network is based on a concatenated alignment of the 16S ribosomal RNA (rRNA) gene, *gapA*, *gyrB*, *pyrH*, *recA*, and *rpoA* spanning 5,473 nt positions. Arrows indicate relative placements according to listed strains. Scale bars are relative to the given panels and represent the number of base substitutions per site. **(B)** Details on *Aliivibrio sifiae*, *Aliivibrio* sp. “friggae,” and *Aliivibrio wodanis* groups. **(C)** Detailed view of “magnii” and “thrudae” clades. **(D)** Strain details for the “bragi” and “vili” clades. See Figure 3, for details on *Aliivibrio fischeri, Aliivibrio logei*, *and Aliivibrio salmonicida*.

To analyze the evolutionary variance within species and the average evolutionary distance between species, strains were assigned to designated groups based on the network model (Figure 1). Briefly, the concatenated gene loci dataset was imported as a nucleotide dataset into MEGA X (Kumar et al., 2018; RRID:SCR_000667) version 10.1.7. Fourteen groups were assigned by the Sequence Data Explorer in MEGA using *P. luminescens* subsp. *laumondii* TT01^T^ as outgroup and *Aliivibrio* sp. appey-12 as a singular group. Distance estimation with SE was calculated for within and between groups of species under the conditions: uniform rates among sites and pairwise deletion was used while applying the JC69 as substitution model. All estimations were statistically tested with 1,000 bootstrap replications. MEGA was further used to construct a Neighbor-joining tree using the MLSA, 16S rRNA, *gapA*, *gyrB*, *pyrH*, *recA*, *rpoA*, concatenated *recA-rpoA, gyrB-rpoH*, and *gyrB-recA* with equivalent parameters as given for the distance measurements. Resulting newick files from each inferred tree was compared topologically against the MLSA using the MutualClusteringInfo algorithm in the TreeDist R package (Smith, 2020). Due to conflicting overlap between sequences in the 16S rRNA alignment, *Photobacterium angustum* strains ATCC 33977 and S14 were removed from the datasets only prior to tree construction and topological comparison.

Sequence identities between and within designated groups were estimated as described for evolutionary distances using single genes as well as the MLSA and a 5-gene concatemer excluding the 16S rRNA gene. The python script identity.py^1^ was created to calculate sequence identities. In short, the script evaluates all sequences pairwise, removing any gapped positions before calculating the identity as a percentage. For each dataset (single genes and MLSA) the identity values were enlisted in either of two subsets; those associated with the same genus (within-genera) or any different genera (between-genera). The same procedure was repeated to differentiate subsets of intra- and inter- species identity values. Values not within and between hitherto described species were filtered. Distributions were plotted as log_10_ transformed histograms to simplify identification of overlapping data. Subsets became colored based on their affiliation as intra- or inter- subsets. GC content was calculated using Biopython GC (Cock et al., 2009; RRID:SCR_007173).

## Results and Discussion

### Phylogeny of *Aliivibrio* Reveals 12 Distinct Species Clades

In this study, a MLSA scheme based on six concatenated genes (16S rRNA gene, *gapA*, *gyrB*, *pyrH*, *recA*, and *rpoA*) were used to infer the phylogeny and evolutionary relationships in the *Aliivibrio* genus. Based on the MLSA sequence data both the inferred phylogenetic network (Figure 1) and phylogenetic tree (Supplementary Figure 1) remained congruent with only minor topology inconsistencies. Twelve individual clades were identified of which seven corresponded to clades described in earlier studies: *A. fischeri* (Urbanczyk et al., 2007), *A. finisterrensis* (Beaz-Hidalgo et al., 2010), *Aliivibrio* sp. “thorii” (Ast et al., 2009), *A. sifiae* (Yoshizawa et al., 2010), *A. wodanis* (Lunder et al., 2000), *A. logei* (Bang et al., 1978), and *A. salmonicida* (Egidius et al., 1986). The results corroborate the reclassification by Urbanczyk et al. (2007), the wider description of *Aliivibrio* (Ast et al., 2009) and confirm the presence of *Aliivibrio* sp. “thorii,” *A. finisterrensis* and *A. sifiae* to the genus. Sixteen strains could not be affiliated to any of the described clades. These strains gave rise to one singular branch (appey-12) and five clades in which we suggest the provisionally names “friggae,” “magnii,” “thrudae,” “bragi,” and “vili” (Figure 1) in order to provide working names in line with other species within *Aliivibrio* genus that have derived their names after Norse mythology gods (*A. wodanis*, *A. logei*, *A. sifiae,* and *Aliivibrio* sp. “thorii”). Clades inferred by the neighbor-joining approach (Supplementary Figure 1) had, except for *Aliivibrio* sp. “magni” and *Aliivibrio* sp. “thrudae,” robust support values. The friggae clade consists of five strains and includes the SR6, SA12 and ATCC 15382, previous classified as wodanis (Ast et al., 2009), EL58, and A.H1309/4.1. These strains were isolated from different hosts such as fishes (Atlantic salmon and Pacific cod), gorgonian coral and bobtail squids. The “thrudae” clade represent three strains isolated from lumpfish while the “magnii” clade represent two isolates from amphipods. Filtered seawater is the source of all three strains composing the “vili” clade. Lastly, strains in the “bragi” clade originate from skin ulcer and head kidney samples isolated from Atlantic salmon.

### In-Depth Analysis of Species Groups and Distances

**Figure 2:**
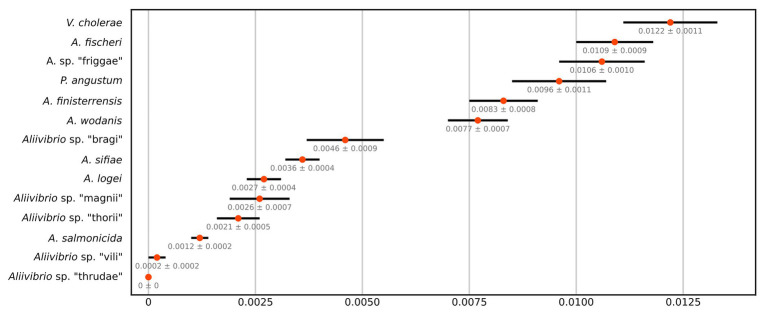
JC69 corrected intra-species distances of the MLSA dataset as number of base substitutions per site. Orange centroids reflect the average over all sequence pairs compared within each group and are drawn with SE values from 1,000 bootstrap replicates.

This study utilizes several strains from each species group in *Aliivibrio* rather than a single representative type strain. This approach provides a more robust statistical measure of group affiliations (intra-species), their circumference, and observed interrelations between groups (inter-species). The results showed fluctuating evolutionary variances in *Aliivibrio*, *Vibrio*, and *Photobacterium* (Figure 2) that closely reflect the circumference of clades in the phylogenetic tree (Supplementary Figure 1). *Aliivibrio* species ranged from the narrow intra-species variance of *A. salmonicida* (0.0012) to the nine times wider variance of *A. fischeri* (0.0109). Still, these measurements are expected to be inaccurate for clades represented by a low number of strains such as *Aliivibrio* sp. “vili” and *Aliivibrio* sp. “thrudae.”

**Figure 3:**
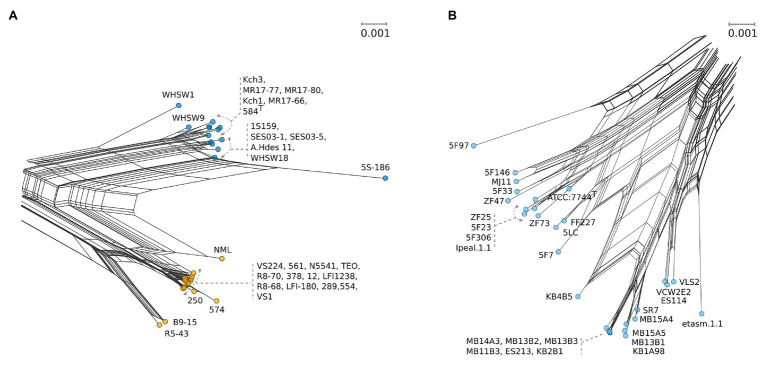
Detailed selections from NeighborNet Figure 1 with uniform scales showing number of base substitutions per site. **(A)** Strain details for *A. logei* (blue) and *A. salmonicida* (yellow). **(B)** Detailed view for the *A. fischeri* clade (pale blue).

Analysis of inter-species distances using the JC69 substitution model is shown in Table 1. These values reflect the distance and error value deviation between species groups as they appear in the phylogenetic tree diagram (Supplementary Figure 1). In *Aliivibrio*, the average evolutionary inter-species distance was 0.060 where the general defined species groups diverged by ≥0.041. The most distant species groups were between *A. fischeri* and *Aliivibrio* sp. “bragi” (0.104), while the smallest distance was between *A. salmonicida* and *A. logei* (0.013), which make them the closest neighboring species in *Aliivibrio* (Figure 3A). It is noteworthy that the low sequence variances of *A. salmonicida* and *A. logei* contrast the number of different host species. Extended sampling of environments might result in the emergence of one interchangeable species rather than two, as a full genome ANI approach has reported representatives of both *A. salmonicida* and *A. logei* as “s__Aliivibrio salmonicida_A” (Parks et al., 2020). This is intriguing as *A. salmonicida* causes cold-water vibriosis and *A. wodanis* causes wodanosis and/or winter ulcer in Atlantic salmon, while *A. logei* is not known to be a salmon pathogen. The phylogenetic structure of *A. fischeri* reflects differences in the host species, but also colonization behavior (Bongrand et al., 2016). Comparable to the *A. fischeri* phylogenetic clades, *A. salmonicida*, *A. wodanis*, and *A. logei* similarities may relate to colonization effectiveness while their differences may be related to behavioral specialization and dependent on environmental factors such as temperature (Nishiguchi, 2000; Hatje et al., 2014; Sunagawa et al., 2015). When considering the 5-gene average GC content (Table 2), higher values (42.5–43.1%) were measured compared to the reported genome average of *A. salmonicida* (39.8%), *A. wodanis* (39.4%) and *A. fischeri* (39.0%; Hjerde et al., 2008, 2015; Califano et al., 2015). Indeed, the genome traits of *A. salmonicida* are suggestive of host specificity adaptation (Hjerde et al., 2008), which do not rule out that other lineages of *Aliivibrio* may have evolved by similar ecological strategies. Genetic drifts for specialization of Aliivibrios could involve genes with lower GC content than the house keeping genes, such as the MLSA scheme used in this study.

**Table 1:** JC69 corrected inter-species distances (lower) of the MLSA dataset as number of base substitutions per site with SE values (upper) from 1,000 bootstrap replicates.

	Group	1	2	3	4	5	6	7	8	9	10	11	12	13	14	15	16
1.	*A. finisterrensis*		0.004	0.004	0.004	0.004	0.004	0.004	0.004	0.004	0.004	0.004	0.003	0.004	0.006	0.008	0.006
2.	*A. fischeri*	0.073		0.004	0.004	0.004	0.004	0.004	0.004	0.004	0.004	0.004	0.004	0.004	0.006	0.008	0.006
3.	*A. logei*	0.086	0.100		0.001	0.003	0.003	0.003	0.003	0.004	0.003	0.003	0.004	0.003	0.006	0.008	0.006
4.	*A. salmonicida*	0.085	0.097	0.013		0.003	0.003	0.003	0.003	0.004	0.003	0.003	0.004	0.003	0.006	0.008	0.006
5.	*A. sifiae*	0.078	0.095	0.050	0.051		0.003	0.003	0.003	0.003	0.003	0.002	0.003	0.003	0.006	0.008	0.006
6.	*Aliivibrio* sp. “vili”	0.083	0.101	0.043	0.045	0.046		0.003	0.003	0.004	0.003	0.003	0.003	0.003	0.006	0.008	0.006
7.	*Aliivibrio* sp. “thrudae”	0.077	0.098	0.040	0.041	0.046	0.039		0.003	0.003	0.002	0.002	0.003	0.003	0.006	0.008	0.006
8.	*Aliivibrio* sp. “bragi”	0.088	0.104	0.041	0.041	0.047	0.040	0.041		0.004	0.003	0.003	0.003	0.003	0.006	0.008	0.006
9.	*Aliivibrio* sp. appey-12	0.082	0.104	0.067	0.067	0.050	0.065	0.051	0.064		0.003	0.003	0.004	0.003	0.006	0.008	0.006
10.	*Aliivibrio* sp. “magnii”	0.083	0.101	0.046	0.045	0.050	0.038	0.031	0.036	0.056		0.003	0.003	0.003	0.006	0.008	0.006
11.	*Aliivibrio* sp. “friggae”	0.076	0.096	0.053	0.053	0.036	0.048	0.030	0.047	0.042	0.037		0.003	0.002	0.006	0.008	0.006
12.	*Aliivibrio* sp. “thorii”	0.062	0.093	0.065	0.065	0.046	0.053	0.056	0.059	0.072	0.062	0.051		0.003	0.006	0.008	0.006
13.	*A. wodanis*	0.080	0.097	0.056	0.058	0.041	0.052	0.044	0.052	0.050	0.047	0.030	0.055		0.006	0.008	0.006
14.	*P. angustum*	0.156	0.148	0.164	0.164	0.165	0.164	0.161	0.163	0.169	0.163	0.161	0.165	0.161		0.008	0.006
15.	*P. luminescens*	0.262	0.253	0.262	0.260	0.262	0.262	0.260	0.260	0.264	0.262	0.259	0.260	0.260	0.259		0.007
16.	*V. cholerae*	0.177	0.166	0.186	0.185	0.184	0.184	0.188	0.183	0.190	0.186	0.186	0.186	0.183	0.185	0.239	

Calculated average between *Aliivibrio* groups was found to be 0.060. Inter-species identity values are additionally provided in Supplementary Table 3.

**Table 2:** Minimum sequence identities for *Aliivibrio* from trimmed and ungapped sequence pairs within intra-species groups.

Group	Strains	MLSA identity (%)	16S identity (%)	5-gene identity (%)	5-gene GC (avg, %)
*A. finisterrensis*	12	≥ 98.30	≥ 99.74	≥ 97.76	43.134
*A. fischeri*	30	≥ 97.88	≥ 98.80	≥ 97.44	42.898
*A. logei*	14	≥ 99.19	≥ 98.80	≥ 99.14	43.088
*A. salmonicida*	18	≥ 99.45	≥ 99.66	≥ 99.39	42.647
*A. sifiae*	19	≥ 97.77	≥ 99.46	≥ 97.25	42.502
*Aliivibrio* sp. “vili”	2	≥ 99.96	100	≥ 99.95	42.917
*Aliivibrio* sp. “thrudae”	3	100	100	100	42.894
*Aliivibrio* sp. “bragi”	3	≥ 99.54	99.83	99.46	42.742
*Aliivibrio* sp. “magnii”	2	≥ 99.74	100	99.67	42.778
*Aliivibrio* sp. “friggae”	5	99.71–98.17	≥ 98.72	99.74–98.00	42.679
*Aliivibrio* sp. “thorii”	3	99.96–99.49	99.83–97.87	≥ 99.93	42.537
*Aliivibrio wodanis*	22	≥ 98.11	≥ 99.66	≥ 97.62	42.653
*Aliivibrio*	134	≥ 89.42	≥ 96.65	≥ 87.05	42.786

The MLSA void of the 16S rRNA gene is here defined as a 5-gene concatemer. *Aliivibrio* sp. appey-12 is included in the full set of *Aliivibrio*. For individual gene measurements see Supplementary Table 4.

### Assessment of Sequence Identity Suggests Improvements in Operational Taxonomic Assignment to *Aliivibrio* Using the *pyrH* or *rpoA* Marker

**Figure 4:**
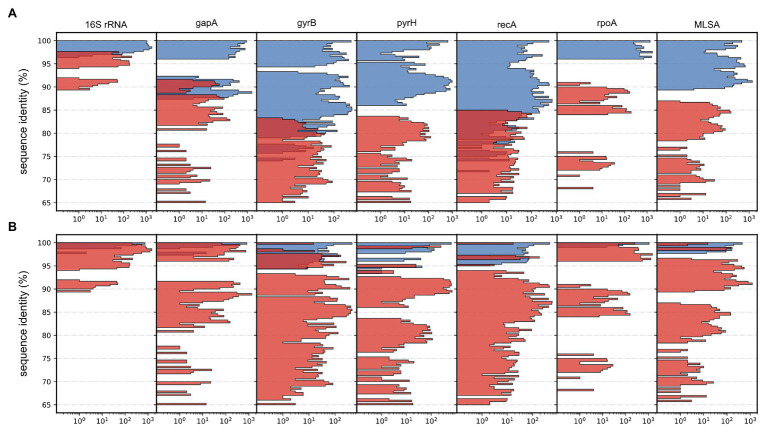
Histograms depicting distributions of pairwise sequence identities for each applied gene and the multilocus sequence analysis (MLSA). Data counts (*x*-axis) are log10 transformed for improved visualization and presented as colored subsets based on relative affiliation. **(A)** Between-genera (red) and within-genera (blue). **(B)** Intra-species (red) and inter-species (blue).

Gene sequence identity is frequently used as distance measurements in homolog comparisons and for clustering operational taxonomic units (OTUs; Edgar, 2017). Here, the inferred phylogeny of *Aliivibrio* was measured interchangeably for species and genera. Estimates based on the 16S rRNA gene sequences show ≥98.80% intra-species identity and ≥96.65% for *Aliivibrio* (Table 2). Similarly, Urbanczyk et al. (2007) reported ≥97.4 intra-species identity among four representative *Aliivibrio* species, illustrating the limited resolving power that corroborate previous assessments by Sawabe et al. (2013). Sequence identity showed no overlap between the within-genera and between-genera subset for the genes *rpoA*, *pyrH*, and the concatenated MLSA (Figure 4A). Gaps between subsets of *rpoA*, *pyrH*, and MLSA were 5.31, 2.45, and 2.72%, respectively. The two extremes of *rpoA*; *P. angustum* ATCC 25915^T^ and *A. sifiae H1-2* (between genera) shared 90.67% identity, while *Aliivibrio* sp. ATCC 15382 and *A. fischeri* 5LC (between species in same genera) shared 96.01% identity. Hence, the *rpoA*, *pyrH*, and MLSA datasets have capability to discriminating genera, but dataset overlaps are unreliable at species level differentiation (Figure 4B). Genus-level overlap was observed for the 16S rRNA gene (1171 bp, covering hyper variable regions v3–v8). This causes potentially inaccurate or erroneous classification and OTU clustering of Aliivibrios using near full length 16S rRNA gene sequences. Utilization of the MLSA dataset resulted in lower intra-species identity (≥97.77%) and considerably reduced identity for the whole *Aliivibrio* genus (≥89.42%), indicating improved resolution. Also, the 5-gene concatemer, without the 16S rRNA gene, resulted in low identity values (Table 2). However, estimates for *A. logei* and *Aliivibrio* sp. “thorii” were found contradicting, suggesting the 16S rRNA gene to be favorable for classification of some species.

### Reasonable Phylogenetic Resolving Power in Either *recA*, *pyrH*, or *gyrB* Demonstrate a Potential to Classify Aliivibrios Similarly as the Full MLSA Tree

**Figure 5:**
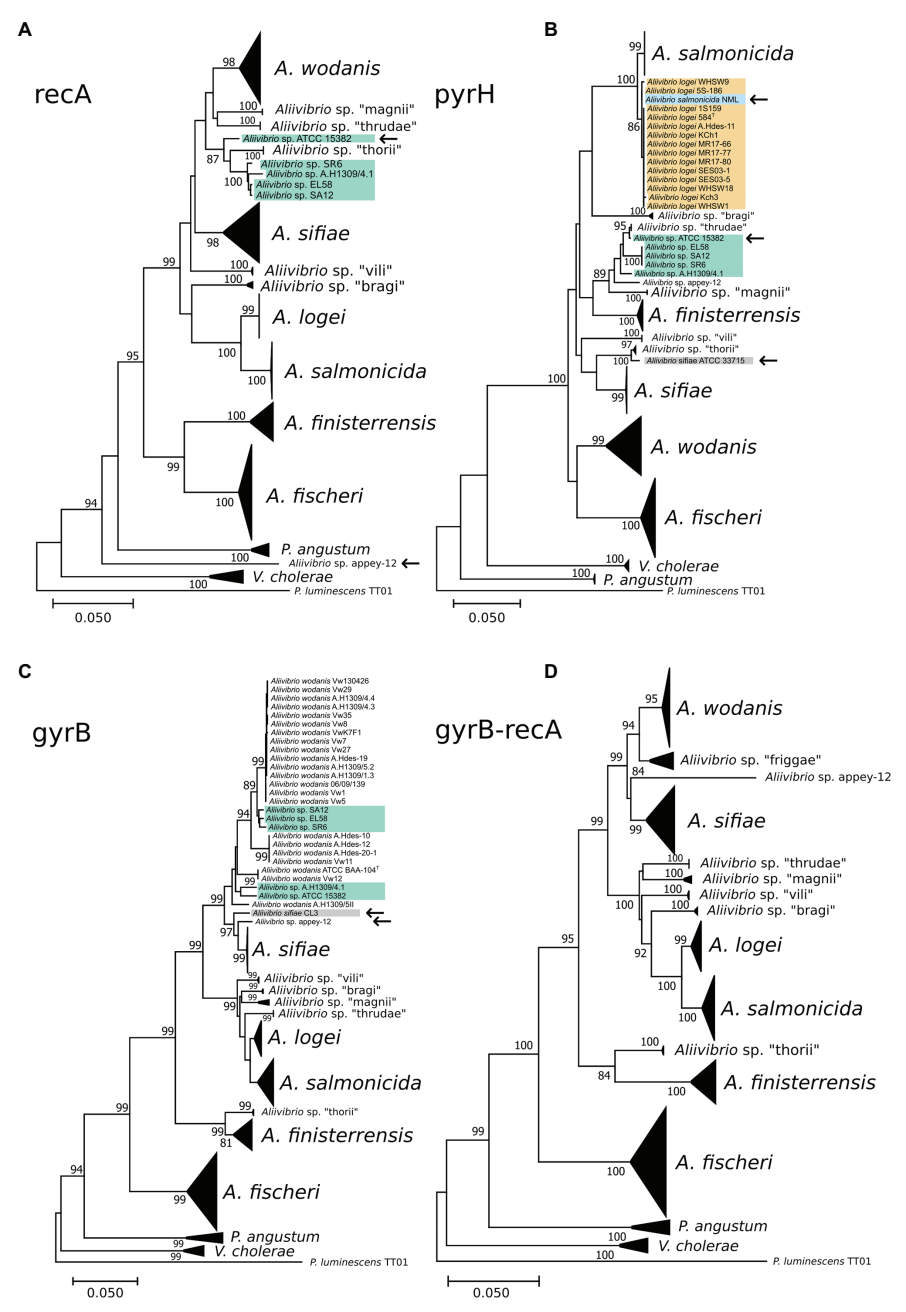
Phylogenetic reconstruction of the marker genes *recA* **(A)**, *pyrH* **(B)**, *gyrB* **(C)**, and *gyrB-recA* **(D)**. Collapsed clades represent conserved classification similarly as in the full MLSA tree. Strains interfering in neighboring or other clades are marked by an arrow and strains of *Aliivibrio* sp. “friggae” has teal background color.

The ability of individual gene markers to classify monophyletic groups with shared topology to the MLSA scheme was assessed. Marker gene *recA* produced a polyphyletic group of *Aliivibrio* sp. “thorii” and *Aliivibrio* sp. “friggae” (indicated in Figure 5A) that shared 67% of the MLSA topology with a misplacement of *Aliivibrio* sp. appey-12. Similar ability to resolve species was observed by *pyrH* (sharing 56.66% of the MLSA topology) in which individual strains of *Aliivibrio* sp. “friggae,” *A. sifiae* and *A. salmonicida* mixed with neighboring clades (Figure 5B) – similar to previously reported *pyrH* discrepancies in the *Vibrio* group (Pascual et al., 2010). Marker *gyrB* shared 73.51% of the MLSA topology. Manual assessment of the tree revealed *A. wodanis* and *Aliivibrio* sp. “friggae” as polyphyletic (Figure 5C) while *Aliivibrio* sp. appey-12 interfere with the *A. sifiae* clade. Still, *gyrB*, *pyrH*, and *recA* markers show significant improvement for *Aliivibrio* classification compared to the 16S rRNA, *gapA*, and *rpoA* which had 54.66, 53.73, and 53.73% topological similarity to the MLSA, respectively. Visual inspection of the resulting trees from 16S rRNA and *gapA* (Supplementary Figures 2, 3) show concerns in resolving *A. sifiae*, *A. logei* and smaller clades like *Aliivibrio* sp. “thorii.” Furthermore, the relative wide identity gap between genera described earlier becomes apparent for the *rpoA* marker tree (Supplementary Figure 4). It firmly discriminates *Aliivibrio* from *Vibrio* and *Photobacterium*, but show similar conserved nature as 16S rRNA for highly similar strains like *A. salmonicida* and *A. logei*.

Although, none of the discussed markers showed discriminatory power equal to that of the MLSA, paired combinations of concatenated *gyrB*, *pyrH*, *recA*, and *rpoA* marker sets were tested. Comparable classification to the MLSA was found in *recA-rpoA* (data not shown), *gyrB-pyrH* (data not shown), and *gyrB-recA* (Figure 5D). Based on mutual clustering information trees from these markers shared 68.17, 77.45, and 80.46% of the MLSA topology, respectively. High topological similarity by *gyrB* produced a discriminating power of *gyrB-recA* that best resembled the MLSA phylogeny for *Aliivibrio*.

## Conclusion

In this study, using 143 strains we have applied MLSA to gain new insight into the evolutionary structure and relationships in the *Aliivibrio* genus. Five new clades (friggae, vili, magnii, thrudae, and bragi) and one singular branch was identified in addition to the seven earlier described clades. These presented clades can be illustrated as a snapshot of current knowledge using available data. Future sampling will likely be expanding the complexity and number of clades in genus *Aliivibrio*.

In this study the discrepancy in intra-species variance for some clades, highly identical sequences may be attributed to a bias toward singular sampling origins or repetitive sampling of target species. The different *Aliivibrio* clades, independent of host range, show different inter-species sequence variances but may also be globally distributed with little sequence variations. This underline the need to include a sufficient number of strains that represent the population for each species, and not only type strains in taxonomic studies.

Host-associated microbiomes can influence their host’s welfare and health and there is an ongoing effort to identify individual members and their contributions. To help, insight into the evolutionary structure of a genus would be beneficial. Here, the MSLA scheme generated is successfully used to infer a significant insight into the evolutionary relationships in the *Aliivibrio* genus. As a major member of the *Vibrionaceae* family in marine environments (Sunagawa et al., 2015; Machado and Gram, 2017), it includes members pathogenic to aquaculture species. Therefore, awareness is required to reinforce phylogenetic relationship of strains within the genus *Aliivibrio*. Continuing the MLSA approach would be of great value to further investigate the distribution and prevalence in the marine environment and to improve the accuracy of clinical diagnosis when *Aliivibrio* is detected from farmed aquatic animals. The identification of *Aliivibrio* species determined by the *gyrB-recA* approach could be an alternative and even more cost-effective way for a rapid and informative molecular method down to the taxonomic level of species within the *Aliivibrio*.

## Data Availability Statement

The datasets presented in this study can be found in online repositories. The names of the repository/repositories and accession number(s) can be found at: https://www.ncbi.nlm.nih.gov/, PRJEB34882.

## Author Contributions

TK conducted and performed the bioinformatic analyses. NW coordinated the work. TK and CK drafted the manuscript. TK, CK, and NW authors provided critical feedback and helped shape the research, analysis, and manuscript. All authors contributed to the article and approved the submitted version.

### Conflict of Interest

The authors declare that the research was conducted in the absence of any commercial or financial relationships that could be construed as a potential conflict of interest.

## Footnotes

^1^github.com/tkl014/aliivibrio_identity
