# Supplementary material for: Phylogenetic Revision of the Genus Aliivibrio: Intra- and Inter-Species Variance Among Clusters Suggest a Wider Diversity of Species
Source: Front Microbiol. 2021 Feb 18;12:626759. doi: 10.3389/fmicb.2021.626759 (PMC7930494; doi:10.3389/fmicb.2021.626759)
Supplement: Supplementary file 2 [file Data_Sheet_2.docx]

Supplementary Material

## Supplementary Table 2. Summary of 53 sequenced and assembled genomes in which sequence data for the MLSA was obtained from in this study. Genome coverage was calculated based on successfully mapped reads and total achieved assembly length.

|  | Reads | Scaffolds | N50 | Assembly length | Coverage |
| --- | --- | --- | --- | --- | --- |
| Maximum | 8,428,252 | 1,435 | 211,881 | 5,196,236 | 503.40 |
| Minimum | 448,642 | 91 | 3,731 | 3,737,369 | 27.40 |
| Average | 3,806,489 | 343 | 62,646 | 4,438,554 | 247.43 |

**Supplementary Table 4.** Minimum sequence identity measurements for each inferred species group and gene. Results are based on trimmed and ungapped sequence pairs within each group. Strain *Aliivibrio* sp. appey-12 is included in the full set of *Aliivibrio*.

| Species | Strains | gapA identity (%) | gyrB identity (%) | pyrH identity (%) | recA identity (%) | rpoA identity (%) |
| --- | --- | --- | --- | --- | --- | --- |
| *A. finisterrensis* | 12 | ≥ 99.48 | ≥ 94.35 | ≥ 98.21 | ≥ 96.71 | ≥ 99.75 |
| *A. fischeri* | 30 | ≥ 99.22 | ≥ 94.62 | ≥ 97.75 | ≥ 95.86 | ≥ 99.68 |
| *A. logei* | 14 | ≥ 98.96 | ≥ 98.07 | ≥ 99.50 | ≥ 99.12 | ≥ 99.76 |
| *A. salmonicida* | 18 | ≥ 99.86 | ≥ 98.24 | ≥ 99.17 | ≥ 99.75 | ≥ 99.89 |
| *A. sifiae* | 19 | ≥ 97.41 | ≥ 97.57 | ≥ 93.63 | ≥ 95.48 | ≥ 99.14 |
| *Aliivibrio* sp. "vili" | 2 | 100 | ≥ 99.92 | 100 | ≥ 99.87 | 100 |
| *Aliivibrio* sp. "thrudae" | 3 | 100 | 100 | 100 | 100 | 100 |
| *Aliivibrio* sp. "bragi" | 3 | 98.57 - 98.57 | 99.92 | 99.67 | 99.37 | 99.57 |
| *Aliivibrio* sp. "magnii" | 2 | 100 | 98.83 | 100 | 100 | 100 |
| *Aliivibrio* sp. "friggae" | 5 | ≥ 99.74 | 99.24 - 95.81 | ≥ 97.35 | 99.87 - 96.74 | ≥ 99.57 |
| *Aliivibrio* sp "thorii" | 3 | 100 | ≥ 99.92 | ≥ 99.67 | 100 | 100 |
| *A. wodanis* | 22 | ≥ 99.74 | ≥ 95.64 | ≥ 94.87 | ≥ 96.24 | ≥ 99.25 |
| *Aliivibrio* | 134 | ≥ 87.57 | ≥ 74.25 | ≥ 86.06 | ≥ 71.79 | ≥ 96.01 |


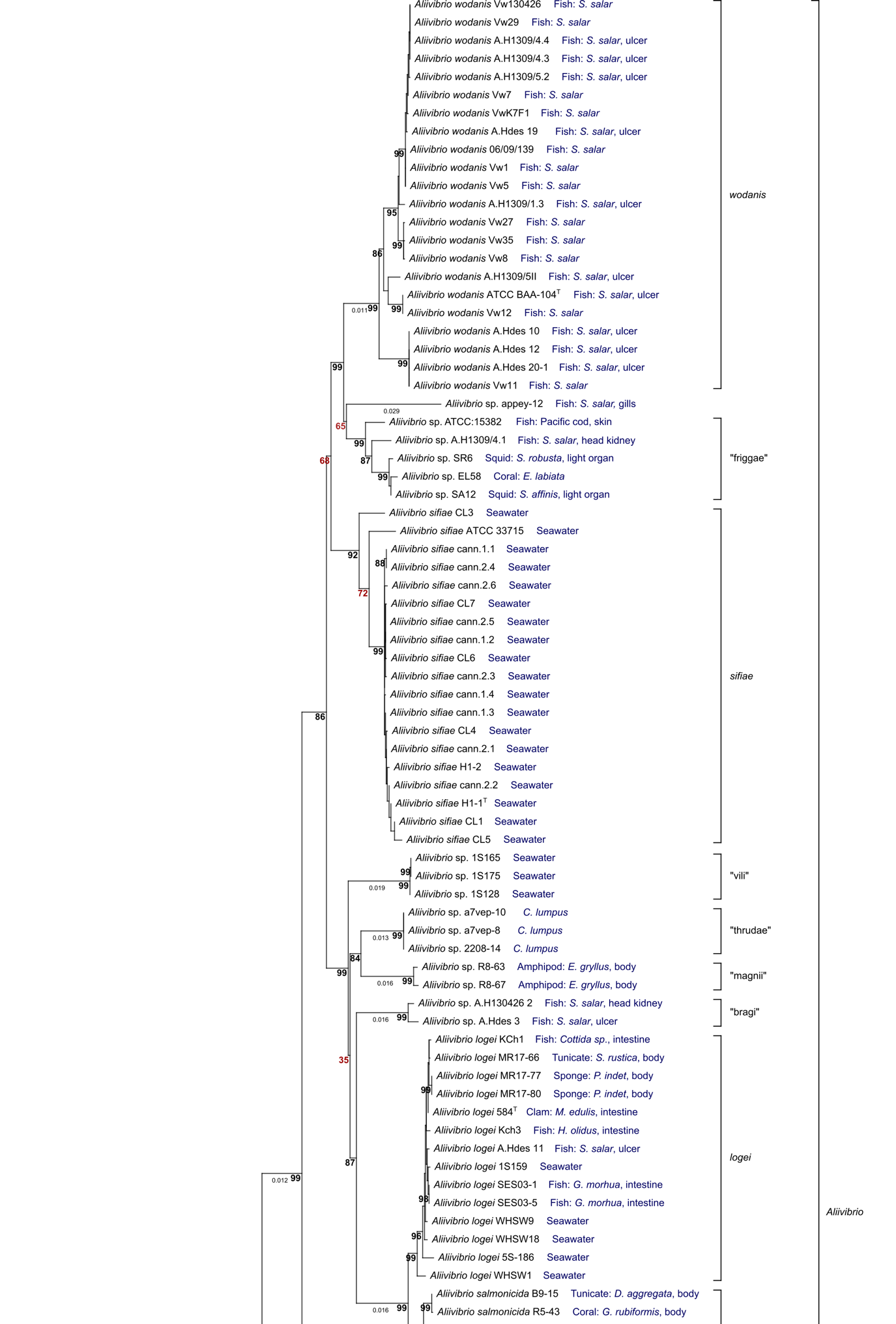


**
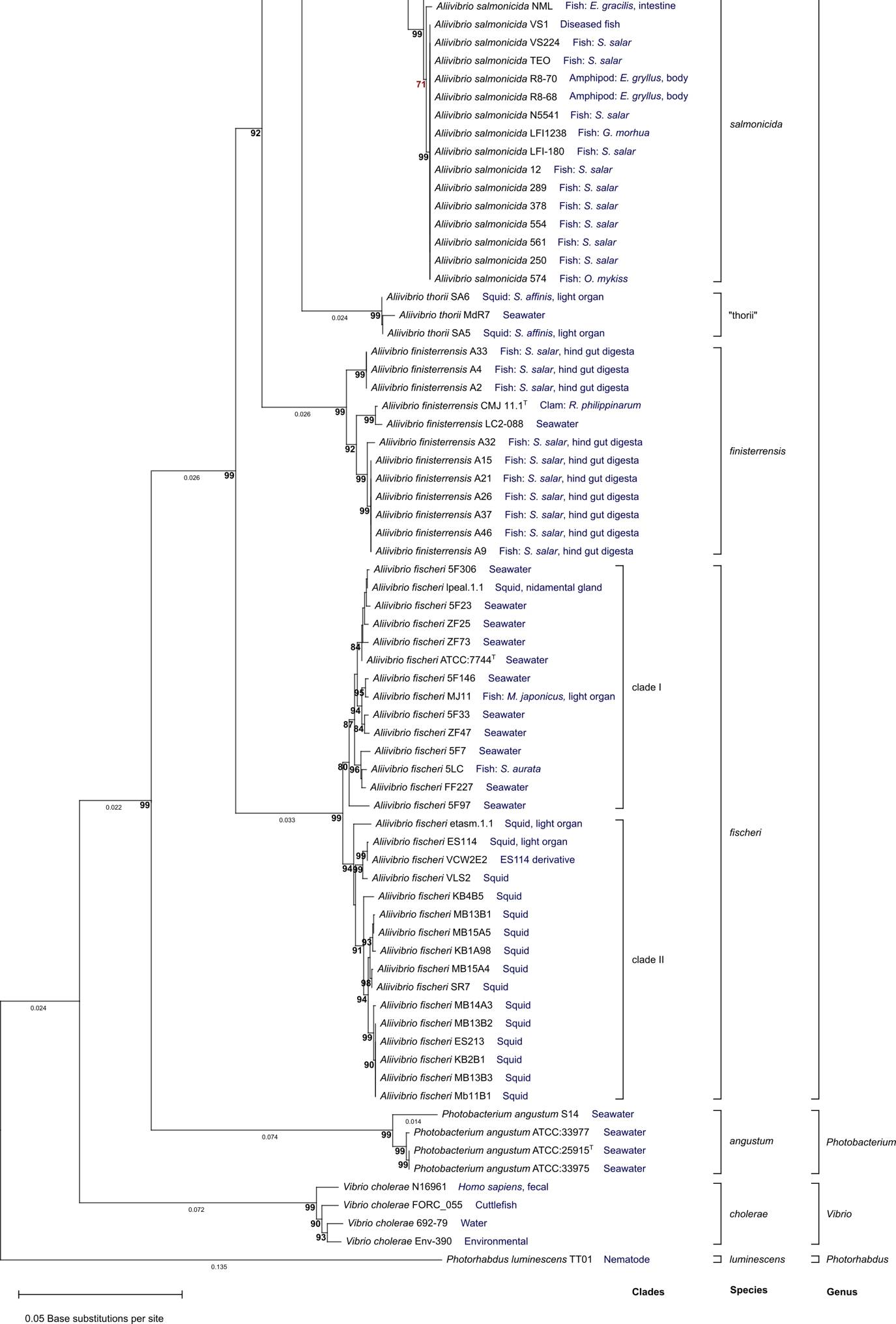
**

**Supplementary Figure 1.** Phylogenetic tree of *Aliivibrio* including with adjacent strains samples of *Photobacterium* and *Vibrio* using *P. luminescens* as an outgroup. Sample source information append leaf nodes (blue text). The tree was inferred from on the aligned MLSA dataset including the 16S rRNA gene, *gapA*, *gyrB*, *pyrH*, *recA* and *rpoA* using Neighbor-Joining*,* ranging 5473 bases. Nodes represent bootstrap support from 1000 replicates. Sum of branch lengths = 0.8737. Support values lower than 80 and branches values shorter than 0.01 are not shown unless of significance as red numbers.


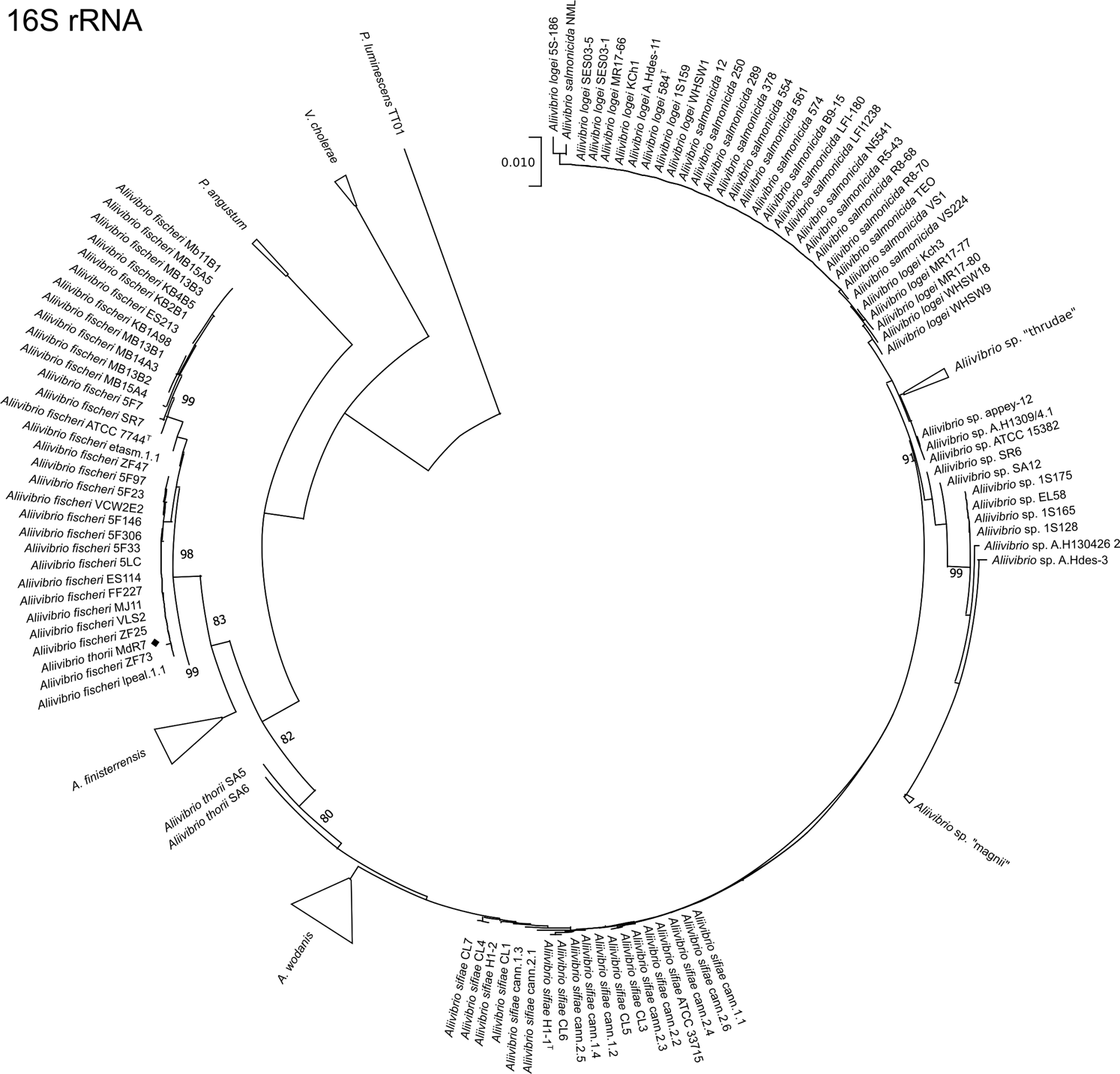


**Supplementary Figure 2.** Phylogenetic reconstruction using the 16S rRNA marker gene in *Aliivibrio*. Compressed clades represent similar classifications as obtained in the phylogeny using the full MLSA. The tree shows statistical values above 80%.


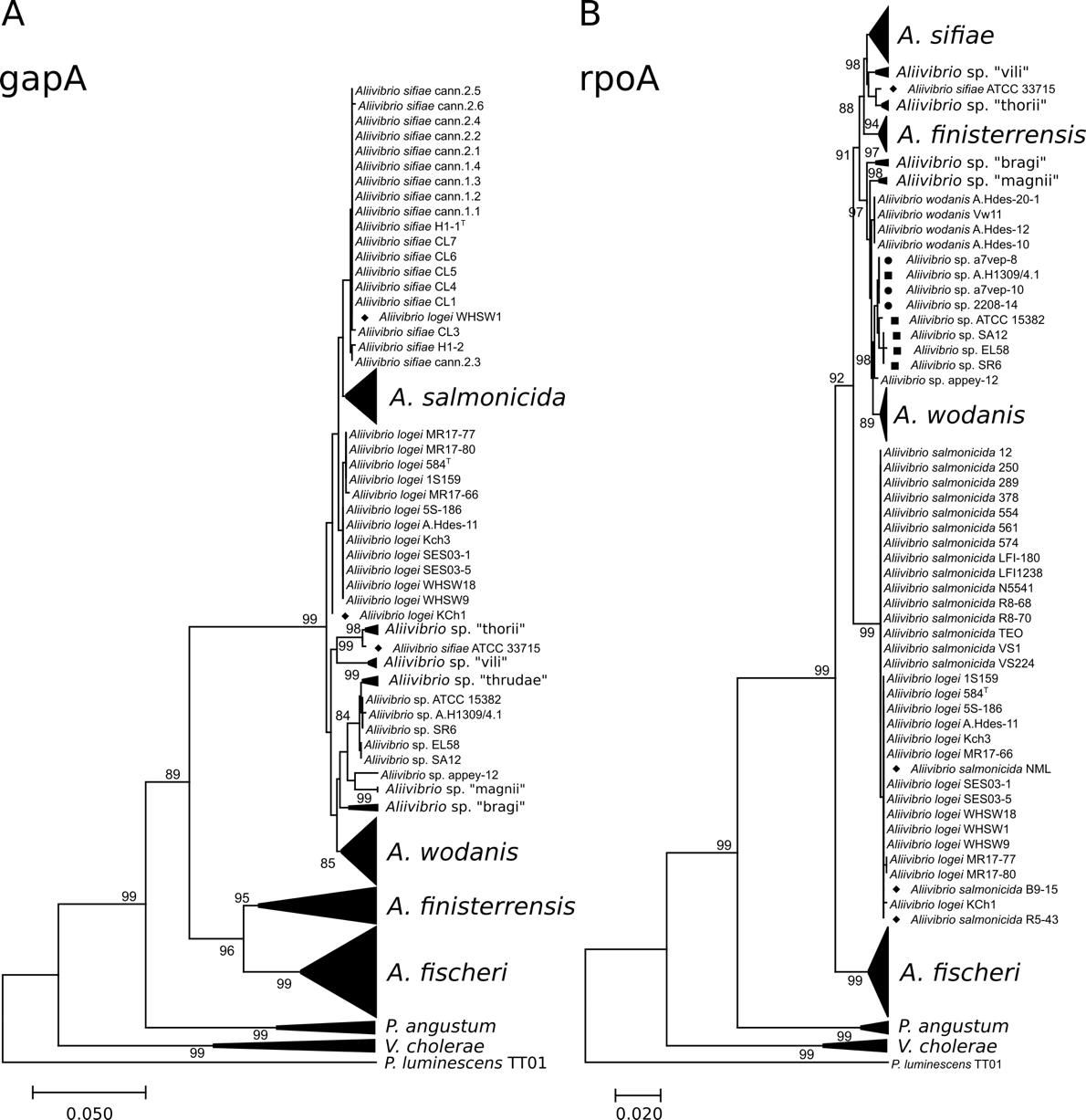


**Supplementary Figure 3.** Phylogenetic trees based on the *gapA* and *rpoA* marker genes. Compressed clades represent similar classifications as obtained in the phylogeny using a full MLSA. Diamond markers indicate strains that interfere with neighboring clades. Square markers in the *rpoA* tree indicate strains belonging to *Aliivibrio* sp. “friggae” and circular markers indicate strains of *Aliivibrio* sp. “thrudae”. Statistical values above 80% are shown.
